# A Hypoxia–Decidual Macrophage Regulatory Axis in Normal Pregnancy and Spontaneous Miscarriage

**DOI:** 10.3390/ijms25179710

**Published:** 2024-09-08

**Authors:** Xu Huang, Zhi Lin, Zi-Meng Zheng, Jia-Lu Shi, Ke-Yu Lu, Jia-Rui Wang, Ming-Qing Li, Jun Shao

**Affiliations:** 1 Laboratory for Reproductive Immunology, Hospital of Obstetrics and Gynecology, Fudan University, Shanghai 200080, China; 23211250009@m.fudan.edu.cn (X.H.); 22211250034@m.fudan.edu.cn (Z.L.); 2 Department of Gynecology, Hospital of Obstetrics and Gynecology, Fudan University, Shanghai 200010, China; shijialu1113@163.com (J.-L.S.); ryanwong0119@163.com (J.-R.W.); 3 Department of Reproductive Immunology, The International Peace Maternity and Child Health Hospital, School of Medicine, Shanghai Jiao Tong University, Shanghai 200030, China; zmzheng24@126.com; 4 Xing Lin College, Nantong University, Nantong 226236, China; lkysomebody123@163.com

**Keywords:** hypoxia, decidual macrophage, abortion, retention, polarization

## Abstract

The significance of hypoxia at the maternal–fetal interface is proven to be self-explanatory in the context of pregnancy. During the first trimester, low oxygen conditions play a crucial role in processes such as angiogenesis, trophoblast invasion and differentiation, and immune regulation. Recently, there has been increasing research on decidual macrophages, which contribute to the maintenance of immune tolerance, placental and fetal vascular development, and spiral artery remodeling, to investigate the effects of hypoxia on their biological behaviors. On these grounds, this review describes the dynamic changes in oxygen levels at the maternal–fetal interface throughout gestation, summarizing current knowledge on how the hypoxic environment sustains a successful pregnancy by regulating retention, differentiation and efferocytosis of decidual macrophages. Additionally, we explore the relationship between spontaneous miscarriages and an abnormal hypoxia–macrophage axis, shedding light on the underlying mechanisms. However, further studies are essential to elucidate these pathways in greater detail and to develop targeted interventions that could improve pregnancy outcomes.

## 1. Introduction

Early pregnancy loss refers to spontaneous pregnancy demise during the first trimester. Statistically, approximately 50% of all pregnancies end in miscarriage at the preclinical stages, due to biochemical or implantation failure, with an additional 10–20% of clinically recognized pregnancies resulting in early pregnancy loss [1]. A wide range of intricate factors have been implicated in spontaneous abortion, including infections, genetic factors, endocrine and immunological dysfunction, and anatomical abnormalities [2]. Clinical studies indicate that women who have experienced one miscarriage are at a markedly increased risk of recurrent pregnancy loss [3]. However, the etiology remains unknown in more than 50% of recurrent miscarriages [4].

Decidual macrophages (dMφs), the second largest immune cells in decidual tissues during early pregnancy, are pivotal in maintaining homeostasis at the maternal–fetal interface to ensure a successful pregnancy [5]. DMφs are capable of phagocytosing apoptotic cells and pathogens to prevent the release of self-antigens and the occurrence of inflammation [6]. Furthermore, they secrete diverse cytokines to serve different functions such as decidualization, spiral artery remodeling, trophoblast invasion, and maintenance of immune tolerance [7]. Dysfunction of decidual macrophages has been associated with pregnancy-related complications, including preterm birth, fetal growth restriction, spontaneous abortion, and preeclampsia [8,9,10,11].

Early pregnancy sets up a hypoxic environment at the maternal–fetal interface, which is conducive to crucial events such as embryo implantation, trophoblast invasion and decidualization [12]. As uteroplacental circulation is fully established, the physiological hypoxia at the maternal–fetal interface alleviates, thereby supporting fetal development. Previous studies have revealed that hypoxia promotes the differentiation of trophoblasts into either invasive or proliferative phenotypes in the first trimester [13]. However, an increasing body of research highlights that hypoxia plays a significant role in regulating the retention, differentiation, and efferocytosis of dMφs.

In light of these findings, this review provides new insights into the critical role and underlying molecular mechanisms of the hypoxia–decidual macrophage regulatory axis in normal pregnancy. We further explore the dysregulation of this axis and its potential etiologies linked to spontaneous abortion, thus expanding the scope for developing innovative treatment approaches aimed at future interventions.

## 2. Dynamic Changes of Blood Oxygen Concentration at the Maternal–Fetal Interface during Gestation

The blood oxygen concentration at the maternal–fetal interface undergoes dynamic changes throughout gestation [14]. Initially, the normal oxygen concentration in the fallopian tube and uterus is maintained at approximately 5% and 2% O_2_, respectively. During the first trimester, the maternal–fetal interface is characterized by severe hypoxia (15–20 mmHg or 2–3% O_2_) attributed to the invasion of extravillous trophoblasts and the occlusion of uterine spiral arteries, which impede the influx of maternal blood into the intervillous space [15]. This low-oxygen environment is crucial in maintaining the pluripotency of human embryonic stem cells and promoting proper embryonic development [16,17].

As the spiral artery plug dissolves and maternal blood enters the intervillous space, the hypoxic condition gradually alleviates from around 12 weeks of gestation, leading to the establishment of uteroplacental circulation and an elevation in oxygen concentration to a physiological range of 8–10% (60–76 mmHg) [18]. Studies on in vitro fertilization (IVF) have shown that embryos cultured in 5% O_2_ have higher implantation rates than those cultured in 20% O_2_ [19]. Additionally, oxygen tension regulates placenta growth by influencing whether cytotrophoblasts proliferate or invade; low oxygen levels promote cytotrophoblast proliferation, while higher oxygen levels encourage differentiation into invasive phenotypes [20].

Moreover, since the rate of reactive oxygen species (ROS) generation grows in proportion to oxygen concentrations, hypoxia helps minimize the generation of ROS [21]. ROS can cause DNA damage and mutations, and the developing conceptus has limited capacity to defend against oxidative stress during early development. Hence, restricting the oxygen delivery to the fetoplacental unit during the first 12 weeks of gestation likely serves to preserve a reduced metabolic rate and shield the embryo from free radical-mediated teratogenesis [22].

## 3. The Pivotal Function of Dmφs in Normal Pregnancy

Decidua, composed of decidual immune cells (DICs) and decidual stromal cells (DSCs), along with trophoblasts, constitutes the maternal–fetal interface [23]. Among them, DICs consist of decidual natural killer cells (70%), dMφs (10–20%), T cells (10–20%), and minor populations of dendritic cells, B cells, and nature killer T cells [24]. The crosstalk among these decidual cells orchestrates the formation of the immunosuppressive microenvironment that promotes immune tolerance of the semi-allogeneic fetus, which is indispensable for ensuring a healthy pregnancy [25]. In this review, we highlight the role of dMφs in early pregnancy.

DMφs, similar to macrophages in other tissues, are typically categorized into M1 or M2 subtypes. Decidual M1 macrophages release inflammatory cytokines to eliminate pathogens, whereas M2 macrophages produce anti-inflammatory cytokines, aiding in vascular remodeling and immune suppression at the maternal–fetal interface [26,27]. Shifts in the M1/M2 macrophage ratio occur throughout pregnancy, and disruption in this balance may give rise to pathological conditions, including preeclampsia, spontaneous abortion, and preterm birth [11,28,29].

Jiang et al. identified dMφs subtypes that differ from the traditional M1/M2 classification. They classified dMφs of early pregnancy into three subsets by flow cytometry: CCR2^–^CD11c^low^ (~80%), CCR2^+^CD11c^high^ (10–15%), and CCR2^–^CD11c^high^ (~5%). CCR2^–^CD11c^low^ and CCR2^–^CD11c^high^ macrophages are identified as M2-like, while CCR2^+^CD11c^high^ macrophages are regarded as M1-like. CCR2^–^CD11c^low^ macrophages are evenly distributed throughout the decidua. CCR2^+^ CD11c^high^ and CCR2^–^CD11c^high^ macrophages are located within and near the extravillous trophoblasts (EVTs), where they regulate trophoblasts invasion [30]. Moreover, CD11c^low^ macrophages express high levels of CD209 and CD206, contributing to extracellular matrix (ECM) formation and tissue remodeling. In contrast, CD11c^high^ macrophages express low levels of CD209 and CD206, with enhanced antigen-presenting capacity [31].

DMφs strive to ensure a successful pregnancy by secreting bounds of cytokines, chemokines, angiogenic growth factors, and proteases, thereby regulating trophoblasts invasion, angiogenesis, immune responses, phagocytosis of apoptotic cells, and tissue remodeling [32]. As trophoblasts infiltrate the maternal–fetal interface, dMφs are enriched and express a wide range of cytokines to modulate the proper invasion of trophoblasts, including anti-invasive cytokines like tumor necrosis factor-α (TNF-α) and interleukin-10 (IL-10), as well as pro-invasive cytokines such as interleukin-1β (IL-1β) and interleukin-8 (IL-8) [33,34]. Moreover, M2 macrophage-derived granulocyte colony-stimulating factor (G-CSF) promotes epithelial-to-mesenchymal transition (EMT), migration, and invasion of trophoblast cells via the PI3K/Akt/Erk1/2 signaling pathway [35]. DMφs also support trophoblast proliferation by secreting interleukin-33 (IL-33) and can induce trophoblast apoptosis via the Fas/FasL pathway [36]. As for the tissue remodeling, CCR2^–^CD11c^low^ macrophages produce angiogenic growth factors such as angiopoietin-1 (Ang-1) and Ang-2, which aid in transforming spiral arteries into vessels with larger diameters, thereby enhancing uteroplacental perfusion [37]. Furthermore, matrix metalloproteinases-9 and -7 (MMP-9 and MMP-7), secreted by this subtype, degrade the ECM, facilitating spiral artery remodeling and ensuring optimal fetal growth and development. In addition, dMφs secrete immunosuppressive molecules such as indoleamine2,3-dioxygenase (IDO), IL-10, and transforming growth factor-β (TGF-β), which establish the crosstalk with other decidual immune cells and maintain the maternal–fetal immune tolerance [6]. For instance, increased expression of CD200R on dMφs enhances IDO activity, suppressing T cell activation and promoting Treg differentiation [38]. Programmed death ligand-1 (PD-L1), present on dMφs in early but not term pregnancy, binds to its receptor, programmed death-1 (PD-1), on T cells. This interaction inhibits immune responses by reducing the generation of the interferon-gamma (IFN-γ), thereby protecting the embryo from maternal immune system attacks and preventing immune rejection [39].

Macrophages are equipped with strong plasticity to adapt to various conditions, including cancer and pregnancy. Intriguingly, dMφs share characteristics with tumor-associated macrophages (TAMs). Both dMφs and TAMs are recruited from the peripheral circulation into decidual or tumor tissues. In cancer, TAMs contribute to immune evasion and angiogenesis by altering their metabolism and modulating the tumor microenvironment. Correspondingly, the role of dMφs in protecting semi-allogeneic fetuses and remodeling spiral arteries is crucial for a successful pregnancy [40]. Hypoxia, a common feature in both cancer and pregnancy, significantly influences macrophage function. The proliferation of malignant tumors leads to the formation of hypoxic areas within the tumor, attracting TAMs and inducing a shift towards the M2 phenotype, thereby promoting tumor immune escape and tolerance [41,42]. This process may suggest similar mechanisms by which hypoxia regulates dMφs [43].

## 4. Hypoxia Promotes DMφs Infiltration and Residence

Circulating monocytes migrate towards areas of injury, diseased tissues, or regions with tumor-associated hypoxia under the influence of chemokines, where they subsequently differentiate into resident tissue macrophages [44]. Macrophages tend to accumulate in hypoxic environments, a phenomenon commonly observed in tumors, driven by the combined effects of chemokines and adhesion molecules [45]. Chemokines and their receptors are abundantly expressed at the maternal–fetal interface, creating a dynamic crosstalk network. In human dMφs, CCR2, CCR5, and CCR1 are highly expressed [46]. In the hypoxic microenvironment of early pregnancy, activation of hypoxia-inducible factor-1 (HIF-1α) leads to increased secretion of CCL2 by DSCs and dMφs [47]. CCL2 binds to its corresponding chemokine receptor, CCR2, activating the Janus kinase 2 (JAK2)-p38 mitogen-activated protein kinase (MAPK) signaling pathways, thereby recruiting macrophages and regulating their immune status [48,49]. The secretion of CCL5 by decidual immune cells is elevated under hypoxic conditions as well. The interaction between CCL5 and CCR5 has been reported to strength residency capacity of dMφs [50]. However, the exact downstream molecules need to be explored. It is worth noting that the proportion of CCR2^+^ dMφs is markedly reduced compared to endometrial macrophages, while the infiltration of CCR5^+^ macrophages is significantly increased compared to the endometrium [47]. This switch in chemokine receptor expression suggests that CCL2/CCR2 is involved in initial recruitment of macrophages, whereas CCR5 is associated with subsequent tissue residency [27]. In conclusion, hypoxia within the decidua induces dynamic changes in the chemokine receptor profile, ultimately promoting macrophages’ accumulation.

Vascular endothelial growth factor (VEGF) possesses chemotactic properties towards both monocytes and macrophages. Lewis et al. demonstrated that higher levels of VEGF correlate with an increased prevalence of TAMs in breast cancer [51]. This finding suggests a potential in vivo chemotactic effect of VEGF on macrophages, particularly under hypoxic conditions. In early pregnancy, hypoxia activates HIF-1α, thereby enhancing the induction of VEGF in the decidua. VEGF plays a critical role in regulating CCL2/CCR2 signaling and the expression of adhesion molecules such as intercellular adhesion molecule-1 (ICAM1), ICAM2, and ICAM5, thereby promoting dMφs recruitment and M2 polarization [52].

Additionally, the receptor activator of nuclear factor-κB (RANK) and its ligand, tumor necrosis factor ligand super family member 11 (TNFSF11) or RANKL, serve as critical mediators orchestrating maternal–fetal tolerance [53]. Research indicates that RANK^+^ dMφs are the predominant subtype at the maternal–fetal interface in normal early pregnancy. The RANKL/RANK signaling pathway upregulates the CCL2/CCR2 signaling in DSCs, potentially facilitating the retention of dMφs within the decidua [54]. Moreover, high levels of adhesion molecules such as CD54 (ICAM-1), CD29, CD31, and CD62L are present through RANKL–RANK interactions between DSCs and dMφs [55]. Tang et al. reported that hypoxia enhances the expression of RANK and RANKL in breast cancer cells through the PI3K/Akt-HIF-1α pathway [56]. However, research on the mechanisms of RANK/RANKL^_^mediated macrophage functions under hypoxic conditions at the maternal–fetal interface remains limited.

**Figure 1:**
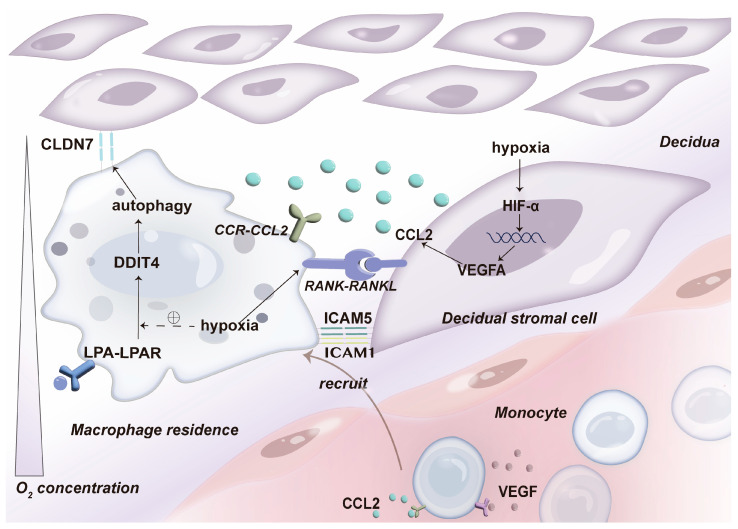
At the maternal–fetal interface, numerous macrophages can be recruited from the peripheral circulation to the decidua under hypoxia. Once recruited, macrophages are retained in the decidua through various adhesion molecules, including ICAM1, ICAM2, ICAM5, and CLDN7.

Claudin 7 (CLDN7), another adhesion molecule localized to tight junctions, is expressed on dMφs [57]. Recently, our research revealed that CLDN7 regulates the adhesion and residency of dMφs through the lysophosphatidic acid (LPA)-autophagy-CLDN7 axis. The accumulation of LPA in dMφs is attributed to active glycerophospholipid metabolism. LPA binding to its receptors, lysophosphatidic acid receptors (LPARs), is crucial for the residency of dMφs [58]. LPA has been shown to upregulate CLDN7 expression in dMφs, while inhibition of LPA receptors significantly reduces CLDN7 levels in dMφs. Our previous experiments demonstrated that supplementation with LPA or the autophagy inducer rapamycin remarkably increases dMφs retention in mice [59] (Figure 1).

## 5. Hypoxia Induces the Polarization of DMφs

The previous statements have described three different subtypes of dMφs. The polarization of these macrophages is similarly susceptible to the dynamic changes in oxygen concentration at the maternal–fetal interface [60]. Relevant research on various consumptive disorders, including malignancies, infections, and tissue damages, has demonstrated that hypoxic environments can lead to the polarization of macrophages into the M2 phenotype [61]. These findings are also applicable to a healthy pregnancy, where the role of M2 macrophages is of fundamental significance.

In response to hypoxia, macrophages experience a metabolic transition that guides their differentiation. During the early stage of pregnancy, the relative lack of oxygen results in the buildup of lactic acid (LA), released from trophoblasts and decidua through aerobic glycolysis [62]. LA has been shown to trigger the M2-like polarization of TAMs through the HIF-1α signaling pathway, independent of the IL-4 and IL-13 pathways. However, Gao Lu et al. were the first to propose that LA generated from trophoblasts exerts bidirectional effects on the differentiation of dMφs, depending on oxygen levels. At 21% oxygen concentration, LA enhances the activity of the SRC kinase, specifically phosphorylating lactate dehydrogenase A (LDHA) and initiating the translational expression of VEGF that ultimately induces the polarization of M2 macrophages. Under oxygen-deficient conditions (1–3% O_2_), however, HIF-1α abundantly upregulated by LA can activate the SRC/LDHA pathway and increase the expression of iNOS to facilitate the transition to M1 [63]. Therefore, as oxygen levels rise from 2% to more than 5% at the maternal–fetal interface during the first trimester, dMφs are inclined to exhibit a mixed profile of M1 and M2 subtypes [64]. ROS generated from nicotinamide adenine dinucleotide phosphate (NADPH), is another key mediator. As ROS heaps up in the deficiency of oxygen, it amplifies the activity of p65 protein, thus driving M1 polarization through the NF-kB pathway [65]. However, the maternal–fetal interface is characterized by a unique metabolic pattern where cells require less oxygen and energy due to the low oxygen atmosphere. Consequently, ROS levels are maintained within a specific range, ensuring a balanced proportion of M1 and M2 for the smooth embryonic development [18].

Fatty acid oxidation (FAO) also acts as the supplement of energy for dMφ polarization. Peroxisome proliferator-activated receptor γ (PPARγ), a critical transcription factor integral to FAO, stimulates the expression of M2 molecular markers [66]. Kolben, T.M. and his colleagues found that the activation of PPARγ suppresses signal pathways such as NF-κB, signal transducer and activator of transcription (STAT), and activator protein 1 (AP-1), promoting M2 polarization. The absence of PPARγ in dMφs during recurrent miscarriages is accompanied by the accumulation of pro-inflammatory cytokines like iNOS, favoring the M1 macrophage phenotype [59,60]. There is evidence that PPARγ expression is impaired under hypoxic conditions in the placenta, but sirtuin1 (Sirt1), a member of the NAD^+^-dependent family of protein deacetylases, can reverse this outcome [67,68]. However, relevant mechanisms of dMφ polarization via the Sirt1/PPARγ pathway remain to be fully elucidated.

With the aid of cytokines, macrophage polarization is also indirectly affected by crosstalk between dMφs and other cells at the maternal–fetal interface. IL-6, a kind of chemokine detected in the trophoblasts, undergoes more obvious upregulation than other factors when macrophages are co-cultured with trophoblasts. After secretion, IL-6 connects with the receptor on the membrane of dMφs, leading to the phosphorylation of the signal transducer and activator of transcription 3 (STAT3), resulting in the transition of macrophages into M2 [69]. Some studies suggest a potential association between IL-6 levels and fluctuation in oxygen concentration. Arbildi P., and Seno K., found that when oxygen levels fell to 5% or lower, the transcription and expression of IL-6 were suppressed [70,71]. Conversely, Sagrillo-Fagundes and his colleagues pointed out that a hypoxia/reoxygenation model, where oxygen levels varied from 0.5% to 8% in vitro, considerably augmented the generation of IL-6. This pattern mirrors the changes in oxygen levels during the first trimester, which may explain why IL-6 intensifies M2 polarization, thereby supporting early pregnancy [72].

Additionally, RANKL, extensively studied in osteoclasts, is observed to be expressed more under hypoxic conditions compared to normoxia, likely due to the elevated amount of HIF-α [73,74,75]. Further studies have demonstrated RANKL is expressed in both trophoblasts and DSCs. In a successful pregnancy, except for its recruitment function, RANKL interacts with RANK to intervene in the Akt/ STAT6 signaling pathway. This interaction enhances the expression of histone H3 lysine-27 demethylase J (Jmjd3)/IRF4, which are necessary for M2 polarization [53] (Table 1).

## 6. Hypoxia Regulates Efferocytosis of DMφs to Maintain Tissue Homeostasis

During embryo implantation, apoptosis is a natural process that facilitates tissue renewal. DMφs, serving as one of the professional phagocytic cells, play an indispensable role in eliminating apoptosis cells (ACs) and other debris, preventing unexpected inflammatory or immune responses [77]. This process is referred to as “efferocytosis”, which is composed of recognition, engulfment, and degradation, three stages involving several signals (“find me”, “eat me”, and “do not eat me” signals) [78]. Current research, though limited, suggests that the mechanisms of efferocytosis may entail the reduction of MHC-II molecules on the surface of trophoblasts, and the modulation of various molecules on macrophages, such as monocyte chemoattractant protein-1 (MCP-1), IL-8 receptors, and ICAM-1 [79]. Following valid efferocytosis, the heightened expression of TGF-β in macrophages leads to a decrease in pro-inflammatory mediators such as TNF, IL-1β, and IL-8 while promoting the release of anti-inflammatory cytokines like IL-10, IL-6, and IL1-Ra, contributing to an immune tolerance state [80]. However, excessive efferocytosis can result in a series of adverse pregnancy outcomes and, in some cases, may be fatal for the embryo.

Efferocytosis of macrophages would be enhanced in oxygen-poor settings. Wang, Y. T. and his team separated resident macrophages from hypoxia-exposed tissues and respectively evaluated their efferocytosis performance under normal oxygen or 1% oxygen conditions. The conclusion was that macrophages in the latter gobbled up a greater number of ACs at a much faster rate, which can probably be illustrated by the shift of metabolism pattern as mentioned above. Macrophages have seemed to engage primarily in a noncanonical pentose phosphate pathway (PPP) during chronic hypoxia. This metabolic adaptation ends up with increased quantities of NADPH, strengthening efferocytosis while minimizing glucose consumption [81]. Interestingly, our recent research yielded conflicting results, suggesting metabolic heterogeneity among macrophages in different tissues. We discovered that efferocytosis in dMφs is more closely associated with glycolysis, regulated by the IL-33/ST2 (IL-1RL1 receptor, also known as ST2) signaling pathway. IL-33, a member of the IL-1 family, is a natural ligand for the membrane-bound IL-33 receptor ST2 (ST2L). Their interaction can downregulate the efferocytosis-related receptor AXL through PI3K/AKT and ERK1/2 pathways. However, soluble ST2 (sST2) serves as a decoy receptor to disrupt the connection between IL-33 and ST2, preventing excessive or insufficient efferocytosis [82]. Ingrid et al. reported a 37.9% increase in sST2 levels from the placenta under hypoxia/reperfusion conditions, while IL-33 levels in the plasma remained unchanged [83]. Hence, it can be hypothesized that efferocytosis at the maternal–fetal interface would become relatively intensified during hypoxia but may remain within normal thresholds through alternative regulatory mechanisms except the IL-33/ST2 pathway.

Another factor worth considering is vasoactive intestinal peptide (VIP), derived from trophoblast cells during early pregnancy. An in vitro experiment showed that monocytes cultured with decidual explants and VIP displayed more intensive efferocytosis compared to those treated with VIP alone, particularly among pregnant donors [84]. This phenomenon may be explained by VIP’s stimulation of IL-10 release from the decidua, as supported by research from Paparini and Gallino [85,86]. However, it remains unclear whether IL-10 activates the guanine nucleotide exchange factor (GEF) Vav1 and the GTPase Rac1 to mediate efferocytosis, as observed in other tissues [87]. Under reduced oxygen levels, FAO rather than glycolysis promotes IL-10 production, further enhancing efferocytosis [88,89].

Apart from the previous discussion, specialized pro-resolving mediators (SPMs) have garnered increasing attention for their role in efferocytosis in hypoxia. Although SPMs are abundant in the placenta, their specific role in efferocytosis of dMφs remains to be elucidated [90] (Table 2).

## 7. Abnormality of Hypoxia-DMφ Regulatory Axis Impels Spontaneous Abortion

Throughout pregnancy, the human placenta experiences significant fluctuations in oxygen levels, which are meticulously regulated to balance the metabolic demands of the fetus and placenta with the risks posed by oxidative free radicals (OFRs) [91]. Spontaneous abortion (SA), often regarded as a typical pregnancy failure, can partly result from an aberrant hypoxia–macrophage regulatory axis. Evidence suggests that hypoxia, characterized by oxygen levels around 1%, is consistently present at the maternal–fetal interface during miscarriages [92].

SA may be exacerbated by alterations in dMφ residency, polarization, and efferocytosis. Regarding macrophage recruitment, our findings indicate that the extent of macrophage infiltration was notably diminished in samples from recurrent miscarriage compared to those from normal pregnancies. Insufficient retention of dMφs may result from abnormal chemokine expression and loss of adhesion molecules, potentially leading to spontaneous miscarriage. In the previous sections, we have discussed the regulatory effects of hypoxia on chemokines and their receptors at the maternal–fetal interface. Notably, serum CCL2 levels are elevated in patients with SA or recurrent pregnancy loss compared to those with normal pregnancies. Under severe hypoxia, excessive CCL2 secretion may induce an overabundance of M1 macrophages, contributing to an inflammatory environment in SA [93]. Conversely, other studies also showed that the expression of DSC-derived CCL8 and serum CCL5 levels are reduced in patients with recurrent miscarriage [94,95]. The reduction of CCL5 and CCL8 may imply weakened CCL5-CCR5 and CCL8-CCR1 signaling, which is essential for the proper positioning of dMφs. Besides, reduced mRNA and protein levels of adhesion molecules, including cadherin 5 (CDH5), selectin E (SELE), selectin L (SELL), ICAM2, and vascular cell adhesion molecule 1 (VCAM1), as well as CLDN7, have been observed in dMφs during SA. Our study highlights the critical role of CLDN7 expression on dMφs in determining pregnancy outcomes. Dysregulation of the LPA receptor–autophagy–CLDN7 axis can result in insufficient retention of dMφs, potentially increasing the risk of spontaneous abortion. Therapeutic studies have demonstrated that supplementation with LPA or the autophagy inducer rapamycin significantly enhances autophagy and retention of dMφs, as well as improving embryo resorption in spontaneous abortion mouse models [59].

In terms of dMφ polarization, it is universally acknowledged that macrophages tend to exhibit the M1 phenotype in spontaneous abortion. Research indicates that M1 macrophages are abundant in the decidua of spontaneous abortions and unexplained recurrent pregnancy loss (RSA), whereas the proportion of M2 macrophages is markedly higher in the endometrium of normal pregnancies. M1 macrophages can release proinflammatory cytokines such as IL-1β and TNF-α, coupled with boosted anaerobic glycolysis and elevated intracellular ROS levels, thus fostering an inflammatory environment [96]. Dysregulation of dMφ polarization may be associated with the disorders of specific molecules [97]. For instance, IL-6 levels were found to be lower in the supernatant of HRT8 cells, imitating SA conditions, which coincided with an increase in M1 macrophages [98]. However, more LA was detected in trophoblasts of SA than those in normal pregnancy. Elevated LA levels may contribute to M1 polarization by activating the HIF-1α/SRC/LDHA pathway [63]. Wang Liling et al. revealed that the protein decorin (DCN) is expressed at higher levels in the decidua of women who suffer an abortion, promoting M1 polarization via the myeloid differentiation primary response 88 (MyD88) /NF-κB signaling pathway.

**Figure 2:**
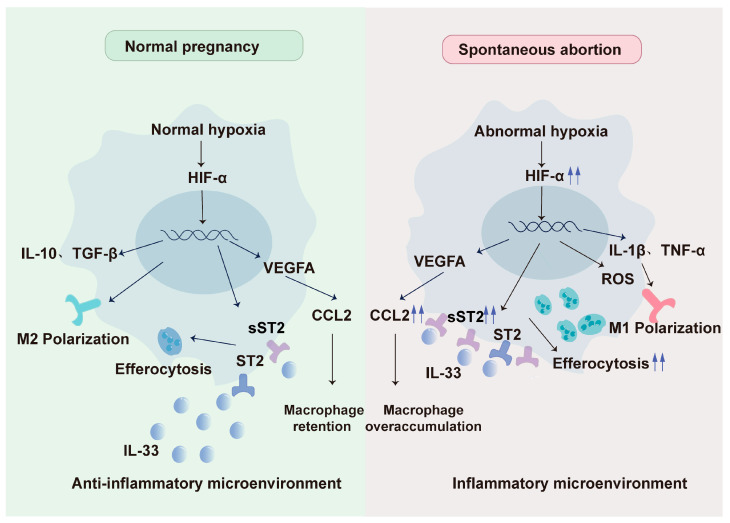
Fluctuations in oxygen levels at the maternal-fetal interface modulate the residency, polarization, and efferocytosis of dMφs, contributing to different pregnancy outcomes. In spontaneous abortion, severe hypoxia (1% O_2_) at the maternal-fetal interface promotes excessive secretion of CCL2 by dMφs, leading to the overaccumulation of dMφs, particularly of the pro-inflammatory M1 subtype. Moreover, the IL-33/ST2 signaling axis is disrupted in dMφs during spontaneous abortion, with elevated sST2 levels exacerbating this imbalance, ultimately driving enhanced efferocytosis.

On the other hand, severe hypoxia in SA may also lead to abnormal efferocytosis, creating a vicious cycle that accelerates and exacerbates abortive outcomes. Sheng Yanran et al. discovered the decrease of IL-33 in dMφs of SA patients, which enhances efferocytosis through the IL-33/ST2 pathway. However, sST2, a negative-feedback regulator, showed an unusual increase due to hypoxia. This increase in sST2 may further amplify the effects of efferocytosis, contributing to faulty placenta formation and immune system disturbances, thereby worsening the conditions leading to SA [89,99] (Figure 2).

## 8. Perspectives

During gestation, there is a significant increase in macrophages within the decidua, an area where hypoxia is a constant presence at the maternal–fetal interface [100]. This hypoxic environment is known to enhance the retention of dMφs, drive their differentiation into the M2 phenotype, and boost efferocytosis through a variety of pathways, which is typical in a healthy pregnancy. However, disruptions to this hypoxia–macrophage regulatory axis can lead to a decrease in dMφ numbers, changes in their differentiation pattern, and an increase in inflammatory responses, potentially leading to miscarriage [59,63]. The precise mechanism by which hypoxia directly influences dMφ retention is still under investigation. It remains to be seen whether specific oxygen concentration levels can dictate the outcome of a pregnancy, distinguishing between normal and pathological conditions. Moreover, there is ample opportunity for innovative approaches targeting the “hypoxia–macrophage regulatory axis” to prevent spontaneous abortion, representing a frontier for future research and therapeutic development.

## Figures and Tables

**Table 1:** The regulation of dMφ polarization under hypoxia.

Molecule	Polarization	Mechanism	Correlation with Oxygen	References
Lactate	M2 M1	Activating SRC-LDHA-VEGF Activating HIF-1α-SRC-LDHA	Inducing M2 polarization in normoxia and M1 polarization in hypoxia conditions	[63]
ROS	M1	Activating NF-kB pathways	Heaps up in the deficiency of oxygen	[65]
PPARγ	M2	Inhibiting NF-κB, STAT, AP-1 pathways	Impaired under placental hypoxia; Sirt1 can reverse this effect	[76]
IL-6	M2	Activating STAT3 pathways	Low oxygen (≤5%) suppresses IL-6; hypoxia/reoxygenation (0.5% to 8%) increases IL-6	[70,72]
RANKL	M2	Intervening in Akt/STAT6 signaling; increasing Jmjd3/IRF4 expression		[53,74]

**Table 2:** The regulation of dMφs efferocytosis under hypoxia.

Molecule	Influence on Phagocytosis	Mechanism	Impact of Oxygen Level on Phagocytosis	References
NADPH	Enhancing phagocytosis	A noncanonical pentose phosphate pathway (PPP) during chronic hypoxia	Strengthened under hypoxia	[81]
IL-33/ST2	Regulating phagocytosis to avoid excessive activity	Controlling phagocytosis via PI3K/AKT and ERK1/2 pathways, inhibiting AXL	Inhibited by sST2 under hypoxia/reoxygenation	[79,82]
VIP	Enhancing phagocytosis	Promoting IL-10 release from decidua, potentially enhancing phagocytosis	Strengthened under hypoxia via FAO	[88,89]
SPM	Enhancing phagocytosis	Potential benefits for phagocytosis		[90]

## Data Availability

No new data were created or analyzed in this study. Data sharing is not applicable to this article.
